# Involuntary movement in stiff-person syndrome with amphiphysin antibodies: A case report

**DOI:** 10.1097/MD.0000000000024312

**Published:** 2021-01-22

**Authors:** Yin-yin Xie, Hong-mei Meng, Feng-xiao Zhang, Buajieerguli Maimaiti, Ting Jiang, Yu Yang

**Affiliations:** a Department of Neurology; b Department of Neurology and Neuroscience Center, First Hospital of Jilin University, Changchun, Jilin, China.

**Keywords:** amphiphysin, involuntary movement, stiff person spectrum disorder, stiff-person syndrome

## Abstract

**Rationale:**

Stiff-person syndrome (SPS) is a rare neurological immune disorder characterized by progressive axial and proximal limb muscle rigidity, stiffness, and painful muscle spasms. Amphiphysin antibodies are positive in approximately 5% of SPS patients. To date, there have been no relevant reports on involuntary movement in cases of SPS with amphiphysin antibodies.

**Patient concerns:**

We describe the case of a 69-year-old man with a 2-year history of progressive stiffness in the neck, bilateral shoulders, and chest muscles, and a more-than-a-year history of dyspnea accompanied by mandibular involuntary movement. The patient was a vegetarian and had good health in the past. The family's medical history was unremarkable.

**Diagnoses:**

He was diagnosed with SPS based on the progressive muscle stiffness, the amphiphysin antibody seropositivity, the continuous motor activity on electromyography, and the effective treatment with benzodiazepines.

**Interventions:**

The patient was orally administered clonazepam and baclofen, and corticosteroid IV followed by prednisone orally.

**Outcomes:**

In the hospital, after treatment with methylprednisolone, clonazepam, and baclofen, the patient's rigidity, stiffness, and dyspnea significantly improved. The involuntary movement of the mandible persisted throughout the treatment process. Currently, under oral treatment with baclofen and clonazepam, the patient's symptoms of muscle stiffness and dyspnea exist, and follow-up is continued.

**Lessons:**

We report a rare and novel case of involuntary movement in SPS with amphiphysin antibodies. The present report explores the relationship between SPS and involuntary movement and expands the spectrum of clinical manifestations of SPS.

## 1 Introduction

Stiff-person syndrome (SPS) is a disabling autoimmune disease of the nervous system characterized by fluctuating muscle rigidity and painful spasms. Recently, with the increasing recognition of the disease, several variants of SPS have been described, leading to the diagnosis of stiff-person spectrum disorder (SPSD), the pathological mechanism of which is not fully elucidated, although it is usually associated with increased autoantibody titers.^[1]^ Amphiphysin antibodies-associated SPS, which is often related to breast cancer and lung cancer,^[2,3]^ is a recognized paraneoplastic syndrome. Reports on amphiphysin antibody-positive SPS combined with involuntary movement are scarce. Here, we report the case of a 69-year-old man with a rare mandibular involuntary movement and amphiphysin antibodies-associated SPS to deepen the understanding of the pathogenesis of the disorder, and expand the pool of known clinical manifestations of SPS.

## 2 Case presentation

A 69-year-old man was admitted to our hospital with a 2-year history of progressive stiffness in the neck, bilateral shoulders, and chest muscles, and a more-than-a-year history of dyspnea accompanied by mandibular involuntary movement. All symptoms disappeared after falling asleep. The patient was a vegetarian and had good health in the past. The family's medical history was unremarkable.

Physical examination on admission showed clear consciousness, fluent language, dyspnea, no obvious positive pathological reflex, and no ataxia. No obvious abnormalities were found in the cranial nerve, sensory, and motor examinations. Mandibular involuntary movement and increased muscle tone in both upper limbs were noted. Bilateral upper limb tendon reflexes were not elicited. He had stiffness in both proximal arms, the neck, the bilateral shoulders, and the chest muscles.

Arterial blood gas analysis showed PCO_2_ 50mm Hg (normal range: 35–48 mm Hg) and PO_2_ 75mm Hg (normal range: 83–108 mm Hg). Initial laboratory studies, including routine blood test, routine coagulation, routine urine test, glycosylated hemoglobin, renal function, liver function, inflammatory markers (erythrocyte sedimentation rate, C-reactive protein, antinuclear antibodies, cytoplasmic antineutrophil cytoplasmic antibodies, and perinuclear antineutrophil cytoplasmic antibodies), and tumor markers, were all normal or negative except for increased homocysteine (32.5 μmol/L; normal range, 0–20 μmol/L) and decreased vitamin B12 levels (59 pg/mL; normal range, 133–675 pg/mL). Serum and cerebrospinal fluid antibodies according to the IIFT method in Beijing Hester Medical Laboratory (including anti-Hu IgG, anti-Ri IgG, anti-Amphiphysin IgG, anti-Tr IgG, anti- glutamic acid decarboxylase (GAD) IgG, anti-Yo IgG, anti-CV2 IgG, anti-ANNA-3 IgG, anti-PCA-2 IgG, and anti-Ma2 IgG) were all negative. Serum and cerebrospinal fluid antibodies according to the BLOT method in Beijing Hester Medical Laboratory (including anti-PNMA2 [Ma2/Ta] IgG, anti-Yo IgG, anti-CV2 IgG, anti-Hu IgG, anti-Ri IgG, anti-Amphiphysin IgG)were all negative except for the anti-Amphiphysin IgG in serum, which was positive. Electroencephalography showed no epileptiform activity. Heart color doppler ultrasound, electrocardiogram, and brain and spinal magnetic resonance imaging were normal. Electromyography (EMG) showed continuous motor activity in the biceps and deltoid muscles when the patient was relaxed. Continuous motor activity disappeared after intravenous injection of diazepam (5 mg). At the same time, the symptoms of dyspnea and muscle stiffness significantly improved. Considering that amphiphysin antibodies-associated SPS is a recognized paraneoplastic syndrome, abdominal color doppler ultrasound and lung computed tomography (CT) were performed, but the results revealed no apparent abnormality. We suggested for the patient to undergo a whole body positron emission tomography-computed tomography; due to economic reasons, the patient refused.

Progressive muscle stiffness, positive anti-amphiphysin antibodies, continuous motor activity in EMG, and effective administration of benzodiazepines all supported the diagnosis of SPS. We administered clonazepam (1 mg/d) and baclofen (5 mg, 3 times a day) orally, and corticosteroid IV (methylprednisolone 80 mg/d, 7 days) followed by prednisone orally (60 mg/d, reduced by 5 mg weekly until discontinued). No antitumor therapy was administered to the patient due to the absence of tumor evidence. Muscle stiffness and dyspnea were obviously relieved. Unfortunately, the patient's involuntary movement of the mandible persisted throughout the treatment process. One year after discharge from the hospital, no obvious abnormality was found in the lung and abdominal CT of the patient. His symptoms of muscle stiffness, dyspnea, and mandibular involuntary movement were still present, being almost similar to the clinical manifestations at admission. At present (approximately 1 year follow-up), the patient takes clonazepam (1 mg/d) and baclofen (5 mg, 3 times a day) orally and the follow-up is continued.

## 3 Discussion

SPS, a rare central nervous system immune disease characterized by axial and proximal limb muscle rigidity, stiffness, paroxysmal painful muscle spasms, and autoimmunity against synaptic antigens (commonly against gamma-aminobutyric acid (GABA)-synthesizing enzyme GAD, especially GAD65),^[2,4]^ has long been considered an inhibitory synaptic transmission disorder.^[5]^ Although GAD65 positivity is very common in patients with SPS, there are also some patients with increased titers of other antibodies, for instance antibodies againstamphiphysin, a protein from the Bin/Amphiphysin/Rvs domain-containing protein family. Amphiphysinis critical for clathrin-mediated endocytosis, which compensates for the rapid exocytosis of neurotransmitters by recycling synaptic vesicles; this is especially important for the high-frequency neurotransmission of GABAergic interneurons.^[1]^ Anti-amphiphysin antibodies interfere with endocytosis in GABAergic synapses, causing a reduction in neurotransmitter release and a diminished GABAergic inhibition in the spinal cord circuitry,leading to clinical manifestations.^[6]^ SPS with amphiphysin antibodies is also called paraneoplastic SPS and is usually associated with tumors, being breast cancer the most commonly associated, followed by lung cancer.^[1]^ Furthermore, paraneoplastic SPS patients show more prominent stiffness in the neck and arms, which is consistent with our patient's manifestations. Considering that abdominal color doppler ultrasound and lung CT revealed no apparent abnormality, we suggested for the patient to undergo whole body positron emission tomography-computed tomography. Unfortunately, due to economic reasons, the patient refused. The patient presented no malignancy after more than 1 year from the diagnosis. We suspect that the case may have a different etiology than that of a paraneoplastic syndrome. In the future, we should conduct more research on patients with similar characteristics to assessour hypothesis.

The different variants of SPS are collectively known as SPSD, and they range in severity from isolated stiff-limb syndromes to progressive encephalomyelitis with rigidity and myoclonus(PERM) or other neurological manifestations.^[7]^ The pathological mechanism of SPSD has not been fully elucidated, but its pathogenesis is usually associated with increased autoantibody titers, including antibodies against extracellular antigens(e.g., GABA-Areceptor,^[8]^ glycine receptor alpha-1 subunit [GlyRα1],^[9]^ dipeptidyl peptidase-like protein 6^[10]^) and intracellular antigens (e.g., GAD65,^[11]^ amphiphysin,^[12]^ and gephyrin^[4]^; Table 1). There is an obvious overlap in the phenotypes of various antibody associated-SPSs, and it is difficult to predict the specificity of antibodies based on the clinical background.^[13]^ Differences in epitope recognition and different areas of expression may explain different symptom spectra. For example, SPS with anti-GlyRs, the most common in patients with PERM,^[14,15]^ always has the clinical feature of parkinsonism.^[7]^ GlyRsare expressed in various regions of the basal ganglia.^[16]^ Piquet et al^[7]^ speculated that GlyRsform another inhibitory mechanism that regulates the function of the basal ganglia, which would explain the manifestation of parkinsonism in SPS patients. The patient in the present case showed mandibular involuntary movement, which has not been reported before, and which may extend the spectrum of clinical manifestations of SPS. We speculate that the reason for the occurrence of involuntary movement is that amphiphysin affects the endocytosis of GABAergic synaptic vesicles and destroys the neural circuits (including direct and indirect pathways) between the basal ganglia and the cerebral cortex, leading to involuntary movements. Further work is needed to clarify the role of amphiphysin antibodies in involuntary movement.

**Table 1:** Illustration about each antibody in SPS including functions of antigen, clinical manifestations and tumor association.

Autoantibodies	Functions of antigen	^∗^Clinical manifestations	Tumor association
Anti-extracellular antigens
GABAaR	Mediate most of the fast GABAergic synaptic actions^[1]^	Status epilepticus, LE^[13,18]^	Thymoma, SCLC, rectal cancer, myeloma^[18]^
GlyRα1	Belong to family of Cys-loop ligand-gated ion channels, which are permeable to chloride and bicarbonate and inhibit postsynaptic cells through hyperpolarization or shunting^[29]^	Hyperekplexia, gait disturbance, myoclonus, parkinsonism or cerebellar signs, brain stem signs, dysautonomia, visual disturbances, seizures, and cognitive decline^[14,15,18,26]^	Papillary thyroid cancer, thymoma, B-cell lymphoma, breast cancer, malignant melanoma, testicular seminoma and SCLC^[15,18,26,27]^
DPPX	A regulatory subunit of the voltage-gated A-type (rapidly inactivating) Kv4.2 potassium channel complex expressed in neuronal dendrites and somatic cells, is the main channel of transient inhibitory currents in central and peripheral nervous system^[30]^	Hyperekplexia, combined with other neurological signs such as cerebellar ataxia,sensory or memory disturbance, behaviour changes, cognitive decline, seizures, brainstem dysfunction, parkinsonism, dysautonomia, pruritus diarrhoea, weight loss^[18,27,31,32]^	B-cell lymphoma^[18,27]^
Anti-intracellular antigens			
GAD65	GABAergic presynaptic proteins and rate limiting enzyme responsible for the synthesis of GABA^[33]^	LE, epilepsy, cerebellar ataxia, temporal lobe epilepsy, oculomotor disturbance, dysautonomia, sensory symptoms^[13,18,27,34]^	Thymoma, lymphoma, breast cancer^[18]^
Amphiphysin	Located intracellularly at GABAergic synapse, which compensates for rapid exocytosis of neurotransmitters by recycling synaptic vesicles, and it is important for high-frequency neurotransmission of GABAergic interneurons^[30]^	Myelopathy and sensory neuropathy ^[35]^	SCLC, breast cancer^[18]^
Gephyrin	Postsynaptically, a large tubulin-binding protein clustering of the 2 inhibitory neurotransmitters (the glycine receptors in the spinal cord and the GABAA receptors in the brain)^[36]^	Gait disturbance, dysarthria and dysphagia^[4,13]^	Mediastinal cancer ^[4]^

Antibodies against neuronal surface proteins may cause pathogenicity through complement activation and inflammatory cytotoxicity, leading to loss of receptors by internalization or to receptor blockage bybinding.^[17]^ Compared with diseases mediated by autoantibodies against neuronal surface targets, those related to autoantibodies against intracellular targetsare generally considered non-pathogenic.^[18]^ However, the manifestations related to amphiphysin antibodies challenge this traditional perception from a clinical and laboratory perspective.^[19]^ The intrathecal injection of amphiphysin antibodies toan SPS mousemodel induces clinical symptoms parallel to those seen in humans.^[1]^ In addition, the passive transference of amphiphysin IgG torats has revealed a dose-dependent nature of the central hyperexcitability phenotype, which may serve clinicians in their daily practice.^[20]^ Werner et al^[19]^ proved that, after passive transfer of amphiphysin antibodies, presynaptic vesicle pools and clathrin-coated intermediates are markedly reduced in rats. Irani^[21]^ proposed several options for where and how the exact antibody–antigen interactions occur. He suggested that antibodies access targets by passing presynaptic boutons, which have a relatively indiscriminate function to uptake antibodies, or by accessing the vesicles that merge with the presynaptic membrane.

The diagnosis of SPS is based on several features: clinical features, supplemented by EMG, that indicate continuous motor unit activity, antibody detection, response to diazepam, and differential diagnosis (including focal and systemic dystonia, hereditary spastic paralysis, motor neuron disease, myelopathy, and tetanus).^[2,22]^ In the present case, the typical EMG feature disappeared after intravenous administration of diazepam, which has not been reported in the past. In the future, this may be an important clinical feature for the diagnosis of SPS.

Due to the rarity of SPSD, evidence to guide treatment decisions is scarce. The purpose of treatment is to relieve symptoms and regulate the autoimmune process.^[23]^ In accordance with the pathogenesis of SPS, GABA-enhancing drugs and immunomodulators can be used. Benzodiazepines are the first line of treatment; they can help inhibit the uninhibited neuronal pathways. Diazepam, as a GABA-A agonist, has muscle relaxation and anti-anxiety properties,^[24]^ and Baclofen, as a GABA-B agonist, can control spasticity.^[25]^ Immunoregulation includes the use of immunoglobulins, glucocorticoids, or plasmapheresis. Studies have shown that SPSs with antibodies directed against cell surface antigens (e.g., GlyR) are responsive to immunotherapy,^[26]^ but that SPSs with antibodies against intracellular antigens (e.g., GAD) are not.^[14]^ Moreover, according to Manhalter et al,^[3]^ corticotherapy has a controversial effect on SPS. Second line immunotherapy(e.g, rituximab andcyclophosphamide) has also been used in recent years.^[27]^ However, a recent placebo-controlled randomized trial of rituximab in SPS patients demonstrated no statistically significant difference between rituximab and placebo.^[28]^ In this case, the symptoms of muscle stiffness and dyspnea were alleviated after treatment with methylprednisolone, clonazepam, and baclofen, but did not fully disappear. Furthermore, the involuntary movement of the mandibula persisted throughout the treatment process.

We report a rare and novel case of involuntary movement in SPS with amphiphysin antibodies. This article highlights the importance of therapeutic trials to confirm the diagnosis of SPS and the understanding of the relationship between SPS and involuntary movement. In addition, it expands the spectrum of known clinical manifestations of SPS.

## Acknowledgments

We would like to thank the patient and her family.

## Author contributions

**Conceptualization:** Yin-yin Xie, Hong-mei Meng, Ting Jiang, Yu Yang.

**Data curation:** Yin-yin Xie, Feng-xiao Zhang.

**Investigation:** Yin-yin Xie, Feng-xiao Zhang.

**Resources:** Yin-yin Xie, Hong-mei Meng, Feng-xiao Zhang.

**Supervision:** Yin-yin Xie, Yu Yang.

**Validation:** Yin-yin Xie, Hong-mei Meng, Feng-xiao Zhang, Buajieerguli Maimaiti, Yu Yang.

**Visualization:** Yu Yang.

**Writing – original draft:** Yin-yin Xie, Yu Yang.

**Writing – review & editing:** Yin-yin Xie, Hong-mei Meng, Buajieerguli Maimaiti, Ting Jiang, Yu Yang.
